# Dr. Thomas R. Russell

**Published:** 1917-12

**Authors:** 


**Dr. Thomas R. Russell,** Vt. 1851, the oldest practicing physician in Wis. died at Oshkosh, Oct. 5, aged 90. He was Surgeon of the 1st Wis. Cavalry in the Civil War.

